# Variable responses to ocean acidification among mixotrophic protists with different lifestyles

**DOI:** 10.1093/ismeco/ycaf064

**Published:** 2025-04-18

**Authors:** Shai Slomka, Jolanda M H Verspagen, Jef Huisman, Susanne Wilken

**Affiliations:** Department of Freshwater and Marine Ecology (FAME), Institute for Biodiversity and Ecosystem Dynamics (IBED), University of Amsterdam, Science Park 904, Amsterdam, 1098 XH, The Netherlands; Department of Freshwater and Marine Ecology (FAME), Institute for Biodiversity and Ecosystem Dynamics (IBED), University of Amsterdam, Science Park 904, Amsterdam, 1098 XH, The Netherlands; Department of Freshwater and Marine Ecology (FAME), Institute for Biodiversity and Ecosystem Dynamics (IBED), University of Amsterdam, Science Park 904, Amsterdam, 1098 XH, The Netherlands; Department of Freshwater and Marine Ecology (FAME), Institute for Biodiversity and Ecosystem Dynamics (IBED), University of Amsterdam, Science Park 904, Amsterdam, 1098 XH, The Netherlands

**Keywords:** ocean acidification, mixotrophy, global change, phytoplankton, bacterivory, mixoplankton, protists, primary production, chrysophytes, Ochromonas

## Abstract

Marine phytoplankton are facing increasing dissolved CO_2_ concentrations and ocean acidification caused by anthropogenic CO_2_ emissions. Mixotrophic organisms are capable of both photosynthesis and phagotrophy of prey and are found across almost all phytoplankton taxa and diverse environments. Yet, we know very little about how mixotrophs respond to ocean acidification. Therefore, we studied responses to simulated ocean acidification in three strains of the mixotrophic chrysophyte *Ochromonas* (CCMP1391, CCMP2951, and CCMP1393). After acclimatization of the strains to treatment with high-CO_2_ (1000 ppm, pH 7.9) and low-CO_2_ concentrations (350 ppm, pH 8.3), strains CCMP1393 and CCMP2951 both exhibited higher growth rates in response to the high-CO_2_ treatment. In terms of the balance between phototrophic and heterotrophic metabolism, diverse responses were observed. In response to the high-CO_2_ treatment, strain CCMP1393 showed increased photosynthetic carbon fixation rates, while CCMP1391 exhibited higher grazing rates, and CCMP2951 did not show significant alteration of either rate. Hence, all three *Ochromonas* strains responded to ocean acidification, but in different ways. The variability in their responses highlights the need for better understanding of the functional diversity among mixotrophs in order to enhance predictive understanding of their contributions to global carbon cycling in the future.

## Introduction

Human activity has caused rapid increases in atmospheric CO_2_ levels and the ocean has absorbed ~30% of all anthropogenic CO_2_ emissions thus far [1, 2]. Marine photosynthetic phytoplankton contribute to the CO_2_ sequestration capacity of the ocean [3] by driving the biological carbon pump, which transports biologically fixed carbon from the euphotic zone to the deep ocean, where it is stored away from the atmosphere [4]. As more CO_2_ dissolves in the ocean surface, the waters acidify and the concentration of carbonate ions decreases [5]. Ocean acidification is already manifested by the current decline in ocean surface pH by 0.11 units compared to preindustrial values [6]. Understanding how ocean acidification impacts the primary producers of the ocean is thus of great importance for our ability to predict the impact and extent of climate change, but achieving this understanding is also a complex task [7].

Thus far, the effects of ocean acidification on plankton growth and physiology have mainly been studied in purely photosynthetic phytoplankton [7, 8]. Furthermore, several studies have investigated effects of ocean acidification on heterotrophic protists such as foraminifera [9, 10] and heterotrophic flagellates [11, 12]. To our knowledge, however, prior to the present study the effects of ocean acidification on mixotrophic protists, a recently recognized group of important players in the plankton community, have been completely overlooked.

Mixotrophic protists are unicellular eukaryotes that are capable of both photosynthesis and phagotrophy [13]. Mixotrophs occupy a large variety of aquatic systems, including the oligotrophic ocean gyres as the largest biomes on the planet, where they constitute a significant proportion of the primary producers [14] and the bacterivores [15]. Mixotrophic species can be divided into different functional groups depending on their phototrophic capacity, with constitutive mixotrophs possessing their own plastids, while non-constitutive mixotrophs either steal plastids from other photosynthetic organisms or host photosynthetic endosymbionts [16]. Mixotrophs can be further categorized based on their primary reliance on either photoautotrophy or heterotrophy and their dependence on both energy acquisition modes (obligate vs facultative mixotrophs [17]). Additionally, the metabolic balance of many mixotrophs has been shown to be dependent on environmental conditions such as light, nutrients, temperature, and prey availability [18–21].

By combining two nutritional modes in one cell, mixotrophs challenge the conventional view that photosynthesis and phagotrophy are performed by different functional groups [22]. The role of mixotrophs in global carbon cycling is likely distinct from that of specialist autotrophs and heterotrophs, as models suggest that mixotrophic nutrition increases the efficiency of the trophic transfer of carbon by shortening the food chain, cutting respirational loss, and using nutrients acquired via grazing on bacteria to support primary production [23, 24]. Furthermore, a recent model indicated that mixotrophy can affect changes in pH during phytoplankton blooms [25]. However, to our knowledge the responses of mixotrophs to changes in ambient CO_2_ concentrations and ocean acidification have not been well studied. It has been hypothesized that increasing CO_2_ levels will reduce the cost of inorganic carbon acquisition for mixotrophs, which may shift their nutritional balance to autotrophic growth [26]; if so, mixotrophs would contribute to enhanced sequestration of carbon under conditions that increase ocean acidification.

Conversely, mixotrophs also have the potential to respond to ocean acidification in the opposite direction by increasing their grazing activity. For example, mesocosm studies showed increased abundance of mixotrophic protists under ocean acidification conditions [27, 28] and study results suggested that this finding might be explained by increased availability of phytoplankton prey. Next to such indirect effects via prey availability, increased grazing rates may also constitute a direct functional response of mixotrophs to ocean acidification, as has been reported for a freshwater mixotrophic protist under acidification conditions [29]. Increased grazing activity of mixotrophs could enhance their release of CO_2_ through increased respiration and thereby diminish the sequestration of ocean carbon. Studying the response of mixotrophs to ocean acidification is thus crucial to our understanding of their net role in the marine carbon cycle—as either sinks or sources for inorganic carbon.

Meta-analyses of published culture experiments have revealed overall positive effects of ocean acidification on growth and photosynthesis of noncalcifying eukaryotic phytoplankton [7, 8]. However, these studies also found large variations in the responses of phytoplankton to ocean acidification. Such variations were found between different taxonomic groups and even within the same species. This variability can be explained by the distinct evolutionary histories of different taxonomic groups and differences in the environmental contexts in which organisms have evolved [7, 8, 30, 31]. The presence of carbon concentrating mechanisms (CCMs) in the majority of eukaryotic phytoplankton [32] further complicates the prediction of phytoplankton responses to ocean acidification. The photosynthetic rates of organisms that possess CCMs might not show major changes with elevated CO_2_, as their RuBisCO [ribulose-1,5-bisphosphate carboxylase/oxygenase] enzymes already operate at saturating CO_2_ conditions, although downregulation of CCMs could save energy and facilitate increased growth rates at higher CO_2_ levels [33]. However, some phytoplankton lack CCMs, such as some members of the *Chlorophyta* and *Rhodophyta* [34]. In particular, work on freshwater phytoplankton indicates that chrysophytes cannot take up bicarbonate and rely on diffusive CO_2_ entry as their only source of inorganic carbon [35]. Hence, chrysophytes are likely to benefit from elevated CO_2_ concentrations.

In this study we examined the responses of three functionally distinct strains of the mixotrophic chrysophyte genus *Ochromonas* to simulated ocean acidification. Chrysophytes have been shown to be numerically important in oceanic systems [36–38] and to include species with variable nutritional strategies [39], with mixotrophic representatives contributing significantly to primary production and bacterivory in oligotrophic ocean regions [40, 41]. Specifically, we investigated two related hypotheses: (i) based on the overall positive growth response of purely autotrophic phytoplankton, the growth of mixotrophic chrysophytes will benefit from ocean acidification, and (ii) due to ocean acidification, mixotrophic chrysophytes will become relatively more autotrophic because elevated CO_2_ concentrations will enable higher carbon fixation rates at similar grazing rates. The three *Ochromonas* strains chosen for this study (CCMP1391, CCMP2951, and CCMP1393) were constitutive mixotrophs isolated from three different marine systems previously shown to have different maximal photosynthetic growth rates [42]. The coastal isolate CCMP2951 and the oceanic isolate CCMP1393 were further characterized as a facultative mixotroph and an obligate mixotroph, respectively. While the CCMP2951 coastal isolate is capable of growing heterotrophically in the dark by phagotrophy of prey, the CCMP1393 oceanic isolate requires both photosynthesis and phagotrophic nutrition for growth [20]. The third strain we studied, CCMP1391, was shown to grow autotrophically in the absence of prey [42], and hence we also termed CCMP1391 a facultative mixotroph. Yet, in contrast to CCMP2951, CCMP1391 showed an obligate dependence on phototrophy [43]. Together, these three strains offered opportunities to investigate the responses of mixotrophic chrysophytes to ocean acidification and to assess potential differences in their responses resulting from different mixotrophic lifestyles.

## Materials and methods

### Algal and bacterial strains

The three *Ochromonas* strains CCMP1391 (isolated from the Sargasso Sea in 1980), CCMP1393 (isolated from the Gulf Stream in 1980), and CCMP2951 (isolated from the coast of Japan in 2003) are closely related [43], but they might represent different species [44]. All strains were obtained from the National Center for Marine Algae and Microbiota (East Boothbay, ME, USA). All *Ochromonas* cultures were grown on modified K medium [45] without silicate and Tris-buffer. The base for the growth medium was 40% natural seawater collected from the North Atlantic (35° 47′ 60″ N 28° 24′ 36″ W, 20-m depth) and 60% artificial seawater. The cultures were kept at 21°C under a 14/10-hour light:dark cycle with a light intensity of 100 μmol photons m^−2^ s^−1^ from an 8-channel LED light source. Prior to the experiment, cultures were treated with antibiotics to achieve axenic conditions by use of a previously described protocol [20]. After the treatment with antibiotics, lack of bacterial growth was verified by inoculation into rich BD Difco^TM^ marine broth. For maintaining mixotrophic growth, the *Ochromonas* cultures were supplemented with heat-killed bacterial prey.

Bacterial prey was prepared from cultures of the marine heterotrophic bacterium *Cobetia marina* DSM4741, obtained from the DSMZ-German Collection of Microorganisms and Cell Cultures (GmbH, Braunschweig, Germany). Bacterial cultures were grown on minimal medium consisting of an artificial seawater base and additions of monosodium phosphate (200 μM), ammonium chloride (3.2 mM), glucose (3.5 mM), Tris–HCL buffer pH 7.5 (4 mM), and a trace metal mix based on the protocol for the L1 medium [46]. Late–exponential phase cultures were heat killed for 2 hours in a 60°C water bath. The heat-killed bacterial cultures were concentrated by centrifugation and resuspended in the algal K medium. Stocks were prepared prior to each experiment and kept frozen until use.

### Experimental treatments and setup

Two experiments of carbonate chemistry manipulation were conducted, the first on the strains CCMP2951 and CCMP1391 (experiment 1) and a second identical experiment on CCMP1393 (experiment 2). Due to suspected contamination of the CCMP1393 culture prior to the start of the first experiment, this culture had to be regrown axenically from stock culture, and thus the CCMP1393 experiment was started immediately after experiment 1. For each strain, two different experimental treatments (with six replicates each) were tested: a low-CO_2_/high-pH treatment, with atmospheric CO_2_ levels representative of concentrations in the late 20th century [47] (~350 ppm, pH ~8.3, referred to as “low CO_2_”), and a high-CO_2_/low-pH treatment, with atmospheric CO_2_ concentrations predicted for the end of the current century by use of high-emission scenarios (e.g. RCP8.5) [48] (~1000 ppm, pH ~7.9, referred to as “high CO_2_”). To achieve the different carbon chemistry treatments, the growth medium was aerated with the target CO_2_ concentrations for at least 48 hours prior to inoculation, using a mixture of CO_2_-free air and air containing 5% CO_2_ in different ratios. CO_2_ concentrations in the air used for aeration were verified daily with an EGM-4 gas analyzer (PP systems, Amesbury, MA, USA). Semicontinuous cultures were inoculated in the aerated media, and kept in 50-ml culture flasks filled to the brim (70 ml) to avoid gas exchange with ambient air. Controls for the carbonate chemistry were added to each experiment by means of one high-CO_2_ and one low-CO_2_–media flask without algal cells, which were maintained in the same manner as the experimental replicates. Temperature and light were kept as mentioned in the previous section. Cultures were maintained semi-continuously, with cell densities kept between 2 × 10^4^ cells ml^−1^ and 4 × 10^4^ cells ml^−1^ by regular transfers into fresh, aerated medium to avoid significant drawdown of CO_2_ due to photosynthetic activity during the growth period. Bacterial prey was supplemented in each transfer to a saturating abundance of 2 × 10^7^ cells ml^−1^. Flow cytometry samples, as well as samples for dissolved inorganic carbon (DIC) and pH measurements were taken every transfer (see sections below). In total 7–8 transfers, corresponding to 7–8 generations and a time period of 3 to 4 weeks under experimental treatments, allowed for full acclimatization (after ~5 generations) prior to measuring any physiological parameters. On the final day of the experiment grazing and carbon fixation assays were performed, and samples were taken for measurement of pigment content (see respective sections below).

### Carbonate chemistry

The carbonate chemistry of the media was monitored in every transfer and on the last sampling day, for all experimental replicates and blank controls. After transfer, the leftover culture of each replicate was used for pH and DIC measurements. The pH was measured using a SenTix® H electrode (Xylem Analytics GmbH, Weilheim, Germany) calibrated with DIN/NIST buffers. DIC samples were filtered through a 0.2-μm syringe filter and stored in gas exchange tight tubes, at 4°C until analysis. All DIC samples were measured in three technical replicates at the end of each experiment with a vario TOC cube (Elementar Analysensysteme GmbH, Langenselbold, Germany). The pH and DIC values of each sample were used to calculate the rest of the carbonate chemistry parameters, namely alkalinity and dissolved CO_2_. Calculations were made using the PCO2SYS python toolbox version 1.8.2 [49] and are summarized in Table S1.

### Flow cytometric cell enumeration

In order to determine transfer days, dilution ratios, and prey abundances, both *Ochromonas* and bacterial prey were enumerated regularly on a CytoFLEX flow cytometer (Beckman Coulter, Brea, CA, USA; see Supplementary Information *Materials and Methods* for details). The samples from the last three transfers were used to calculate growth rates as well as relative chlorophyll fluorescence (see Supplementary Information *Materials and Methods* and Fig. S1 for details).

### Pigment content

Culture samples were filtered onto GF-75 filters (Advantec, Tokyo, Japan) and filters were stored at −80°C until extraction of pigments as previously described [50]. Then we analyzed 70 μl of the pigment extract as previously described [51] on a Shimadzu high-performance liquid chromatography (HPLC) instrument (Shimadzu corporation, Kyoto, Japan) equipped with a Hypersil ODS column (Thermo Fisher Scientific) and a diode array detector. The following pigment standards were used (DHI LAB Products, Hørsholm, Denmark): chlorophyll *a* and *c* (2,3), fucoxanthin, β-carotene, diadinoxanthin, violaxanthin, and zeaxanthin.

### Grazing assays

For grazing assays, fluorescently labeled bacteria (FLB) were prepared from *C. marina* based on a previously described protocol [52] adapted as described in Supplementary Information *Materials and Methods*. For the grazing assay, 5 ml of acclimated culture from each replicate was supplemented with nonlabeled heat-killed bacteria to an abundance of 2 × 10^7^ cells ml^−1^ and spiked with FLB to 5% of the abundance of total bacterial prey. Cultures were incubated under experimental conditions for 30 minutes and samples were taken immediately after the addition of FLB (T_0_) and at the end of the incubation period (T_30_). Samples were fixed with formaldehyde (final concentration 1%), incubated in the dark for 20 minutes, flash frozen in liquid nitrogen, and stored at −80°C until analysis.

For detecting ingestion of FLB by *Ochromonas*, samples from T_0_ and T_30_ of the grazing assays were analyzed on the flow cytometer twice: once using a red fluorescence trigger for detection of *Ochromonas* cells and once using a green fluorescence trigger for detecting the FLB. Uptake of FLB by *Ochromonas* cells was then detected by following the changes in the green fluorescence properties of *Ochromonas* cells over the 30-minute incubation (see Fig. S2 and Supplementary Information *Materials and Methods* for details of flow cytometry and estimation of grazing rates). For estimation of the carbon obtained through grazing, the hourly grazing rates obtained from the assay were multiplied by the prey cellular carbon content of 0.13 ± 0.01 pg C cell^−1^. The latter was determined by measurement of particulate organic carbon (POC) on a Vario EL Elemental Analyzer (Elementar Analysensysteme GmbH, Hanau, Germany), following filtration of bacterial stocks onto precombusted GF-75 glass fiber filters and freeze-drying.

### Photosynthetic carbon fixation

For carbon fixation measurements, the classical method of ^14^C labeling was used [53] with adjustments as described in the Supplementary Information *Materials and Methods*. From each acclimated replicate a sample was taken and distributed over three glass scintillation vials, resulting in 2-ml aliquots. After addition of ^14^C-bicarbonate, the vials from five of the six replicates per treatment were incubated for 1 h at three different light levels: darkness, 50- and 100-μmol photons m^−2^ s^−1^. The three vials of the remaining replicate were incubated for 1 h in darkness, 1 h at a light level of 100 μmol photons m^−2^ s^−1^, and 1 T_0_ measurement with sampling immediately upon addition of the ^14^C–bicarbonate (as a quality control of our ^14^C measurements). Due to the relatively short incubation times of 1 h, measured carbon fixation rates likely resembled gross carbon fixation. However, even over this time frame a significant proportion of freshly fixed carbon can be lost due to respiration [54], potentially resulting in a measurement value between the gross and net rates of carbon fixation.

### Statistical analyses

Statistical analyses were performed using the python SciPy and statannotation [55] packages. Normality was tested using the Shapiro–Wilk test. Equality of variances between compared treatments was tested with Levene’s test. To account for unequal variances between the low-and high-CO_2_ treatments, we used a Welch’s *t*-test throughout the data reported in the manuscript to compare between means. The *t*-statistics, degrees of freedom, and *P* values of all statistical comparisons are presented in Table S2.

## Results

### Growth response to simulated ocean acidification

The two experimental treatments emulated the atmospheric CO_2_ concentrations of the late 20th century (~350 ppm, “low CO_2_”) and the CO_2_ concentrations predicted for the end of this century by high-emission scenarios (~1000 ppm, “high CO_2_”). The *p*CO_2_ in the *Ochromonas* cultures was slightly lower than the imposed pCO_2_ treatment due to CO_2_ uptake by photosynthesis and equilibrated at around 320–360 ppm (pH of 8.3) and 780–890 ppm (pH of 7.9–8.0) in the respective CO_2_ treatments (for detailed carbonate chemistry parameters see Table S1).

**Figure 1:**
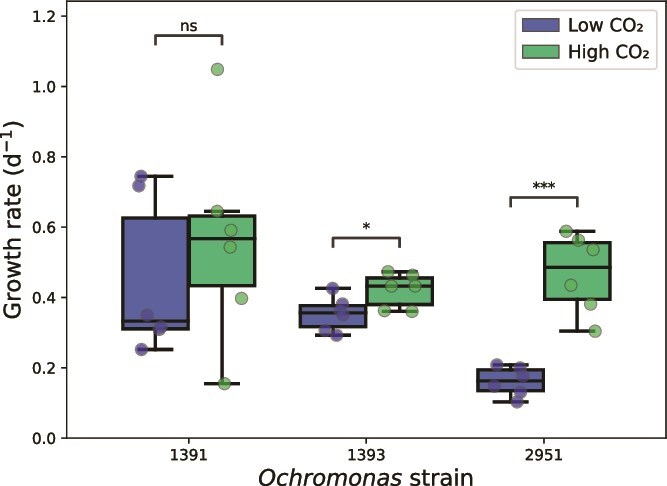
Specific growth rates of the three *Ochromonas* strains in the low-CO_2_ and high-CO_2_ treatments. Asterisks indicate significant differences between treatments (Welch’s *t*-test: ^*^P < .05, ^**^P < .01, ^***^P < .001).

Both CCMP2951 and CCMP1393 had significantly higher specific growth rates in the high-CO_2_ treatment than in the low-CO_2_ treatment (Fig. 1; Welch’s *t*-test, *P* < .001 and *P* < .05, respectively). The strongest growth response was observed for CCMP2951, which increased its growth rate from only 0.16 ± 0.04 d^−1^ (mean ± SD, same as below) in the low-CO_2_ treatment to 0.48 ± 0.11 d^−1^ in the high-CO_2_ treatment. The growth response of CCMP1393 was less pronounced and increased from a mean of 0.35 ± 0.05 d^−1^ in the low- to 0.42 ± 0.05 d^−1^ in the high-CO_2_ treatment. Strain CCMP1391 exhibited a large variability in growth rates between replicates, but did not show a significant growth response, despite higher mean growth rates of 0.56 ± 0.30 d^−1^ in the high-CO_2_ treatment compared to 0.45 ± 0.22 d^−1^ in the low-CO_2_ treatment (Fig. 1).

Two of the three strains, CCMP1391 and CCMP2951, showed a tendency for cell clumping during the experiment, which was further assessed by flow cytometry (see Supplementary Information for details). This analysis revealed that both strains showed significantly more cell clumping with the high-CO_2_ treatment than the low-CO_2_ treatment (Fig. S3; Welch’s *t*-test, *P* < .001 for CCMP1391 and *P* < .05 for CCMP2951). Cell clumping was not observed for CCMP1393. When adjusting cytometric cell counts to account for cell clumping, growth rate responses to the different treatments remained similar (Fig. S4).

### Effects of ocean acidification on the trophic balance of *Ochromonas*

**Figure 2:**
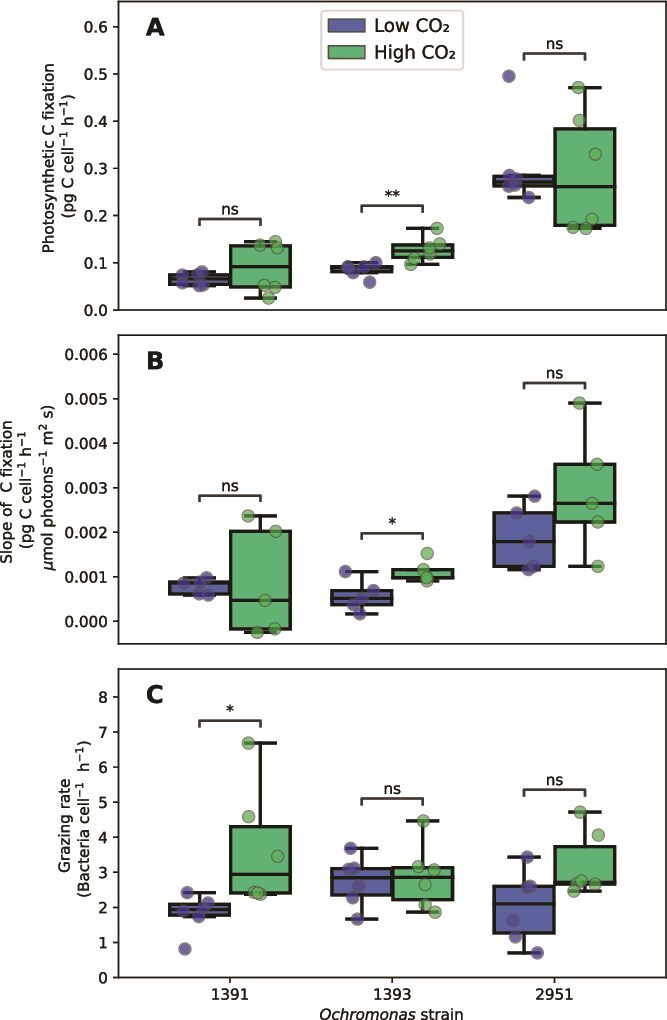
Photosynthetic and phagotrophic rates of the three *Ochromonas* strains in the low-CO_2_ and high-CO_2_ treatments. (A) Carbon fixation rates at an irradiance of 100 μmol photons m^−2^ s^−1^. (B) Slope of the photosynthetic carbon fixation rates versus irradiance, measured between 50 and 100 μmol photons m^−2^ s^−1^. (C) Grazing rates of *Ochromonas*. Asterisks indicate significant differences between treatments (Welch’s *t*-test: ^*^P < .05, ^**^P < .01, ^***^P < .001).

Photosynthetic carbon fixation rates varied between the strains, with CCMP2951 having the highest rates in both treatments, fixing around 0.30 pg C cell^−1^ h^−1^, while CCMP1393 and CCMP1391 exhibited considerably lower rates, which varied between 0.07 and 0.13 pg C cell^−1^ h^−1^. CCMP1393 was the only strain that had a significantly higher carbon fixation rate in the high than in the low-CO_2_ treatment (Fig. 2A; Welch’s *t*-test, *P* < .01). The carbon fixation rate of CCMP1393 also increased more strongly with irradiance in the high than in the low-CO_2_ condition (Fig. 2B; Welch’s *t*-test, *P* = .01). Strain CCMP1391 had a significantly higher grazing rate in the high- than in the low-CO_2_ treatment (Welch’s *t*-test, *P* < .05), while the grazing rates of the other two strains were not significantly affected by the CO_2_ treatment (Fig. 2C).

**Figure 3:**
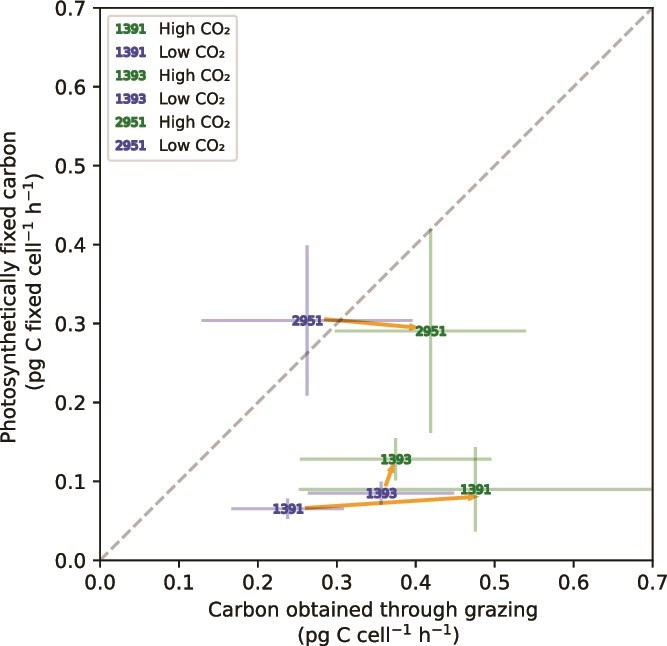
The balance between phototrophic and heterotrophic carbon acquisition for the three *Ochromonas* strains in the low-CO_2_ and high-CO_2_ treatments. The amount of photosynthetic carbon fixed per hour is plotted against the amount of carbon obtained through grazing per hour. Error bars represent standard error of the mean. The dashed grey line is the *y* = *x* line, at which phototrophic and heterotrophic carbon acquisition are equal. Orange arrows are drawn from the low- to the high-CO_2_ treatment of the same strain.

To assess the balance between the two nutritional modes of *Ochromonas*, hourly grazing rates were directly compared to photosynthetic rates via their common currency - carbon. Carbon obtained through grazing accounted for the majority of acquired carbon for strains CCMP1391 and CCMP1393 in both treatments (Fig. 3). CCMP1393 was the only strain to become slightly more autotrophic, increasing the mean contribution of photosynthetic carbon fixation to its total carbon acquisition from 20% to 27%. In contrast, CCMP1391 became even more heterotrophic at elevated CO_2_, increasing the contribution of phagotrophy to its total carbon acquisition from 77% to 82%. Strain CCMP2951 acquired on average approximately half of its carbon from carbon fixation and the other half from grazing, and displayed high variability for both carbon acquisition modes (Fig. 3). Extrapolating the measured hourly rates to daily rates, assuming that photosynthesis is limited to daytime while grazing continues day and night, results in an increasing importance of grazing (Fig. S5). Yet, we cannot tell whether this extrapolation to daily rates is valid, because the assumption of constant grazing rates across the diel cycle might not be met [56].

Thus, in response to elevated CO_2_, CCMP1393 increased its carbon fixation rate and became more autotrophic and CCMP1391 increased its grazing rate and became more heterotrophic, while CCMP2951 did not show a consistent shift between its two nutritional modes.

### Changes in pigmentation in response to ocean acidification

**Figure 4:**
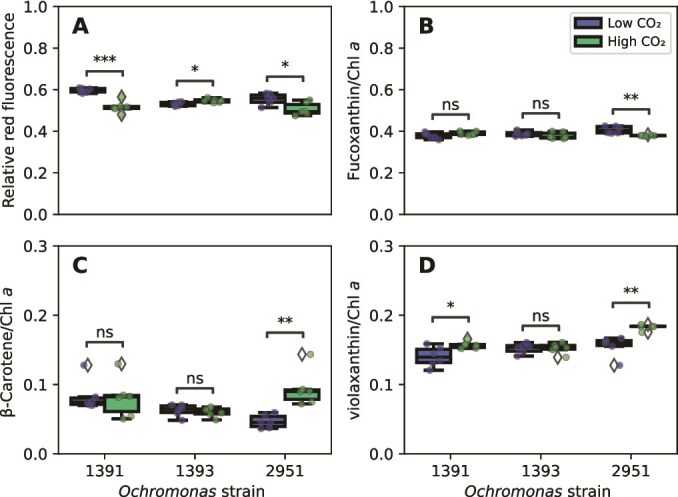
Relative pigment contents of the three *Ochromonas* strains in the low-CO_2_ and high-CO_2_ treatments. (A) Red chlorophyll fluorescence per cell, measured by flow cytometry. (B) Fucoxanthin content, (C) β-carotene content, and (D) sum of violaxanthin and zeaxanthin content (violaxanthin cycle pigments), measured by HPLC and expressed relative to chlorophyll *a*. Asterisks indicate significant differences between treatments (Welch’s *t*-test: ^*^P < .05, ^**^P < .01, ^***^P < .001).

Flow cytometric analysis of the cellular chlorophyll fluorescence revealed significant differences between the CO_2_ treatments for all three strains. CCMP1391 and CCMP2951 decreased their cellular chlorophyll fluorescence in the high-CO_2_ treatment, indicating lower cellular chlorophyll *a* contents (Fig. 4A; Welch’s *t*-test, *P* < .001 and *P* < .05, respectively). This decrease in chlorophyll fluorescence was not linked to changes in cell size, as for both strains, the relative cell sizes based on forward scatter properties of the cells did not significantly change with treatment (Fig. S6). In contrast, CCMP1393 significantly increased its chlorophyll fluorescence in the high-CO_2_ treatment (Fig. 4A; Welch’s *t*-test, *P* < .05). Quantifying the cellular Chl *a* content by HPLC revealed a similar, yet nonsignificant trend as the flow cytometric analysis for CCMP1393 and CCMP2951 (Fig. S7). For CCMP1391 the cellular Chl *a* content measured by HPLC showed an opposite trend to the flow cytometric analysis, with large variability in the high-CO_2_ treatment (Fig. S7).

In strain CCMP2951, the content of the accessory pigment fucoxanthin (expressed relative to Chl *a*) was significantly lower in the high-CO_2_ treatment (Welch’s *t*-test, *P* < .01), while the contents of the photoprotective pigments β-carotene and the violaxanthin cycle pigments violaxanthin and zeaxanthin were significantly higher (Fig. 4B–D) (Welch’s *t*-test, *P* < .01 and *P* < .01, respectively). The violaxanthin cycle pigments were also significantly higher in the high-CO_2_ treatment for CCMP1391 (Welch’s *t*-test, *P* < .05), while CCMP1393 did not show any significant changes in its accessory pigments.

Hence, all three strains altered their pigment composition in response to elevated CO_2_, with one strain (CCMP1393) investing more in photosynthetic pigments while the other two strains invested relatively more in photoprotective pigments.

## Discussion

The resilience of microbes to climate change is critical to the functioning and survival of entire ecosystems [57]. In agreement with our first hypothesis, none of the three mixotrophic chrysophytes investigated in our study was adversely affected by ocean acidification, as all strains either sustained or increased photosynthetic carbon fixation or grazing rates at elevated CO_2_. Moreover, two of the three *Ochromonas* strains (CCMP1393 and CCMP2951) reached significantly higher growth rates in a simulated ocean acidification scenario, thus clearly benefiting from higher CO_2_ conditions. Remarkably, despite the high genetic similarity between the strains, and their overall positive response, only one strain became more autotrophic with increasing CO_2_ concentrations, while the other two strains became either more heterotrophic or did not significantly alter their nutritional balance. Hence, our second hypothesis that mixotrophic chrysophytes will become relatively more autotrophic under elevated CO_2_ conditions was verified for one strain only. The variation in physiological responses among *Ochromonas* strains in this study aligns, however, with variations observed in previous studies on phytoplankton responses to ocean acidification that show that within-genus variability can be high and on par with the variation observed between different genera or functional groups [7, 30, 31]. The strains examined in our study originate from distinct habitats and have different strategies in managing the two carbon acquisition pathways of photosynthesis and grazing [20, 42]. Local adaptation and functional diversity could potentially account for the observed variability in the strains’ responses to ocean acidification.

*Ochromonas* strain CCMP1393 is an obligate mixotroph requiring light and prey in a complementary manner [20]. Its photosynthetic machinery is strongly upregulated by the presence of prey [58]. Strain CCMP1393 exhibited improved growth with high-CO_2_ treatment, coinciding with increased photosynthetic carbon fixation rates. Mean increases of 50% in rates of gross carbon fixation and 20% in growth rate, make it reasonable to assume that much of the growth difference stems from this increase in the carbon fixation rate. The higher carbon fixation and growth rate did not coincide with increased ingestion of prey, and hence higher CO_2_ concentrations facilitated a shift towards relatively more autotrophic growth, even though this strain still obtained most of its carbon from grazing (Fig. 3). Decreased dependence of autotrophic processes on phagotrophy under conditions of higher inorganic carbon concentrations could be an indication that prey-derived carbon serves as a means to alleviate CO_2_ limitations in photosynthetic carbon fixation in obligate mixotrophs, as previously hypothesized for this strain [58].

Strain CCMP2951 is a facultative mixotroph that is able to grow heterotrophically in the dark and decreases its grazing rate with higher light availability [20]. This highly plastic strain showed an almost 3-fold higher mean growth rate in the higher CO_2_ treatment, clearly benefiting from ocean acidification conditions. However, unlike CCMP1393, and against expectations, the source for its surplus energy could not be clearly linked to increased carbon uptake, as the increases in carbon acquisition rates by either phagotrophy or photosynthesis were statistically insignificant. This finding might simply be due to a lack of statistical power, given the observed variability in carbon acquisition rates, or it might also have a more mechanistic explanation, as increased growth despite comparable carbon consumption rates could be possible through increased efficiency in carbon use, e.g. by changes in cell size, decreased respiration costs (for example, decreased photorespiration under conditions of elevated CO_2_) or decreased investment in CCMs and photosynthetic machinery at elevated CO_2_ levels. For example, the decrease in cellular chlorophyll *a* content, observed by both flow cytometry and HPLC (though not statistically significant in the latter) at higher CO_2_ levels could be an indication of a more general downregulation of photosynthetic investments. Because photosynthetic investments contribute a large fraction of the cellular energy requirements in photosynthetic protists [59], their downregulation is expected to decrease cellular energy and carbon requirements and might thus allow for an increase in growth rates. This interpretation is consistent with the observed decrease of the light-harvesting pigment fucoxanthin and relative increase of photoprotective pigments (β-carotene and the violaxanthin cycle pigments) at elevated CO_2_ levels. A similar shift from photosynthetic to photoprotective pigments is commonly observed when phytoplankton are exposed to high light levels and has also been reported for this *Ochromonas* strain when transferred from low to high light conditions [20]. Furthermore, the decrease of light-harvesting pigments that we found for CCMP2951 has also recently been found in other phytoplankton species exposed to elevated CO_2_ [60]. In contrast, CCMP1393 showed an increased cellular chlorophyll content under elevated CO_2_ in line with higher rates of carbon fixation, but did not change its relative pigment composition. This finding suggests large differences in regulation of the photosynthetic machinery between the obligate and facultative *Ochromonas* strains.

*Ochromonas* strain CCMP1391 requires light for growth and can grow autotrophically without prey [42]. Hence, it can be considered a facultative mixotroph, although unlike CCMP2951 it is an obligate phototroph [43]. Yet, strain CCMP1391 shows high grazing rates when prey is available [42] and was the only strain in our experiments not to show a significant growth response to ocean acidification. However, this strain also exhibited large variability in growth rates between replicates in both CO_2_ treatments. Interestingly, both of the facultative mixotrophs, CCMP1391 and CCMP2951, showed higher variance across all physiological assays compared to the obligate mixotroph, suggesting that their higher phenotypic plasticity also resulted in increased variability within these strains. Our grazing assay revealed a significantly higher grazing rate of CCMP1391 in the high-CO_2_ treatment, indicating that this strain became relatively more heterotrophic in response to ocean acidification. Similarly, a previous study on freshwater mixotrophs found increased heterotrophy in more acidic water [29] and raised the possibility that lower pH might facilitate increased motility, as previously observed [61], which results in higher encounter rates with prey. The high variability in growth rates of CCMP1391 make it hard to assess whether the increased grazing rates result in higher growth rates or are negated by, e.g. higher carbon losses and lower carbon use efficiency.

Finally, it is important to note that although we emphasize the differences in CO_2_ concentrations between treatments, pH is an important variable which could also have played a role in shaping the observed responses of the cultures to ocean acidification. pH could be an especially relevant factor in the case of mixotrophs. Combining the contrasting processes of photosynthesis, which increases near-cell pH, with phagotrophy, which has the opposite effect, poses the ability to stabilize surface pH in response to external changes, and has been suggested to be one of the possible benefits of mixotrophic nutrition [25, 62]. Thus, modulation of photosynthetic and phagotrophic rates under different carbonate chemistry regimes could potentially serve as means to maintain pH homeostasis regardless of CO_2_ availability.

In this study, we identified ocean acidification as an important factor that can modulate growth and the nutritional balance of mixotrophs. As ocean acidification comes along with a multitude of environmental changes, it is important to try to predict how all factors interact together to shape the responses of mixotrophs, although this is an extremely challenging task due to the scarcity of available data. Looking at warming effects, for example, only three experimental studies examined the nutritional response of mixotrophic chrysophytes to temperature. Two of these studies used freshwater species [21, 63] and found contrasting results, with one species showing a shift towards heterotrophy with increasing temperature [21] and the other the opposite effect [63]. Yet, an adaptive evolution study conducted on marine *Ochromonas* CCMP1391 and CCMP2951 at different temperatures showed increased growth rates at elevated temperatures for both strains [64]. Considering this finding in combination with the results of our study, where two of the three strains significantly increased their growth rate at elevated CO_2_ levels, we hypothesize that mixotrophic chrysophytes will generally benefit from a future CO_2_-rich and warmer ocean, although there may be differences between short-term and long-term phenotypic responses.

Overall, the three chrysophyte isolates in this study represent just a small fraction of the known and cryptic mixotrophic species and their diverse nutritional strategies. Future expansion of this study to a larger number of mixotrophic species is crucial for a more complete understanding of the effects of ocean acidification on mixotrophs and the consequences of their role in the biological carbon pump, especially considering the variability in responses observed in this study. Further investigation of the mechanisms of responses to ocean acidification could also improve our understanding of mixotrophic physiology and the mechanisms underlying the diversity in energy acquisition strategies found among mixotrophs.

## Supplementary Material

Slomka_etal_SI_ycaf064

## Data Availability

All data generated during this study are included in this published article (and its supplementary information files). Raw data are also available on Figshare (10.6084/m9.figshare.28741820).
