# Supplementary material for: Variable responses to ocean acidification among mixotrophic protists with different lifestyles
Source: ISME Commun. 2025 Apr 18;5(1):ycaf064. doi: 10.1093/ismeco/ycaf064 (PMC12086424; doi:10.1093/ismeco/ycaf064)
Supplement: Slomka_etal_SI_ycaf064 [file slomka_etal_si_ycaf064.pdf]

<u>Supplementary Information for</u>

Variable responses to ocean acidification among mixotrophic protists with different lifestyles

Shai Slomka, Jolanda M. H. Verspagen, Jef Huisman and Susanne Wilken

Department of Freshwater and Marine Ecology (FAME), Institute for Biodiversity and Ecosystem Dynamics (IBED), University of Amsterdam, P.O. Box 94920, 1090 GE, Amsterdam, The Netherlands

Corresponding authors:

Shai Slomka, Department of Freshwater and Marine Ecology (FAME), Institute for Biodiversity and Ecosystem Dynamics (IBED), University of Amsterdam, Science Park 904, 1098 XH,Amsterdam, The Netherlands

s.slomkadeoliveira@uva.nl

Susanne Wilken, Department of Freshwater and Marine Ecology (FAME), Institute for Biodiversity and Ecosystem Dynamics (IBED), University of Amsterdam, Science Park 904, 1098 XH, Amsterdam, The Netherlands

s.wilken@uva.nl

This PDF file contains:

Materials and Methods

Supplementary Figures S1-S7

Supplementary Tables S1-S2

Supplementary Materials and Methods

Flow cytometric cell enumeration

Samples from all replicates were fixed with a mixture of glutaraldehyde (0.25% final concentration) and Pluronic F68 (0.01% final concentration) [1], and were analyzed after a 20-minute dark incubation. For bacterial enumeration, a subsample was diluted 10 times in TE buffer and stained with the nucleic acid stain SYBR Green I (Molecular Probes-Invitrogen, Carlsbad, CA, USA) in a final concentration of 5×10^{-5} of the commercial stock prior to enumeration. *Ochromonas* cells were detected using a red fluorescence (690/50 nm) trigger and gated based on their red chlorophyll autofluorescence and forward scatter properties (see Sup. Figure 1 for an example). Bacterial cells were enumerated using a green fluorescence (525/40 nm) trigger and gated based on their green fluorescence and forward scatter properties. In addition to routine sampling, samples of the cultures before and after each transfer were fixed in the same manner, but were flash frozen in liquid nitrogen and stored at -80 °C for accurate counts and growth rates over each growth period. Those samples were enumerated similarly, with the addition of spiked Fluoresbrite™ Polychromatic Red 2.0 µm Microspheres (Polysciences, Warrnington, PA, USA) as internal standards. The samples from the last three transfers were used to calculate growth rates as well as relative Chlorophyll fluorescence.

The growth rates of all replicates in the two treatments were calculated based on the last three transfers. The growth rate of each replicate for a given growth period between two transfers was calculated as follows:

$$GR_{Transfer} = \frac{\ln(x_{t+\Delta t}) - \ln(x_t)}{\Delta t} \qquad \text{(Eq. 1)}$$

where $x_{t+\Delta t}$ is the *Ochromonas* count obtained by flow cytometric enumeration of the sample after the growth period, x_t corresponds to the count before the growth period, and

Δt is the growth period in days (ranging from one to four days). The growth rate of each replicate was calculated by averaging the replicate's growth rate over the last three transfers.

Cellular chlorophyll fluorescence was analyzed from the flow cytometry samples taken at the end of the last three growth periods. To this end, the mean red fluorescence of the *Ochromonas* population (gate P2 in Supp Figure 1) was divided by that of the red fluorescent beads which were added as internal reference to each sample (gate P1 in Supp Figure 1). For each replicate, these normalized values were then averaged over the last three transfers.

Grazing assays

For preparation of Fluorescently Labeled Bacteria (FLB), a late-exponential bacterial culture was harvested, concentrated by centrifugation and resuspended in PBS buffer (pH=9). The dye 5-(4,6-dichlorotriazinyl) aminofluorescein (DTAF) was added in a concentration of 100 µg per 1 ml culture. The culture was dark incubated with the dye for two hours in a 60°C water bath. The excess dye was then removed by four to five rounds of centrifugation and resuspension in artificial seawater. Final resuspension was done with artificial seawater and aliquots were frozen at -20°C until use. When performing a grazing assay, an FLB aliquot was thawed, sonicated for 10 minutes, and enumerated on the flow cytometer to determine FLB concentration.

For detecting ingestion of FLB by *Ochromonas*, samples from T_0 and T_{30} of the grazing assays were analyzed on the flow cytometer twice – once using a red fluorescence trigger for detection of *Ochromonas* cells and once using a green fluorescence trigger for detecting the FLB. In addition, subsamples from T_0 were stained with SYBR green to verify total bacterial counts at the beginning of the assay using a green fluorescence trigger (as described above). *Ochromonas* cells were identified and gated as described above. Uptake of FLB by *Ochromonas* cells was then detected by following the changes in the green fluorescence

properties of the *Ochromonas* cells over the 30 minutes incubation. The basal green fluorescence of the population was determined based on the T_0 sample. Cells exceeding this baseline green fluorescence were termed "feeding" (see Sup. Figure 2 for an example). The percent of cells feeding at T_{30} was corrected by subtracting the percent of cells feeding at T_0.

The mean number of FLB per feeding cell was estimated based on the mean green fluorescence of the feeding cells ($\overline{GF}_{feeding}$), corrected for basal green fluorescence of *Ochromonas* ($\overline{GF}_{non\ feeding}$), and divided by the mean green fluorescence of the FLB ($\overline{GF}_{FLB}$):

$$FLB\ per\ feeding\ cell = \frac{\overline{GF}_{feeding} - \overline{GF}_{non\ feeding}}{\overline{GF}_{FLB}} \quad \text{(Eq. 2)}$$

The grazing rate (G, in bacteria cell^{-1} h^{-1}) was then calculated as:

$$\mathrm{G} = \frac{feeding\ cells \times FLB\ per\ feeding\ cell}{Total\ Ochromonas \times \Delta t} \times \frac{Total\ Bac}{FLB} \quad \text{(Eq. 3)}$$

where Δt is the incubation period in hours, $Total\ Bac$ is the total bacterial abundance quantified with SYBR Green, and FLB is the FLB abundance.

Photosynthetic carbon fixation

For measurements of carbon fixation, ^{14}C labeled bicarbonate stock of 100 µCi ml^{-1} activity was added to glass scintillation vials containing 2mL aliquots sampled from the experimental cultures (final activity of ~1µCi ml^{-1}). We used six replicate cultures per experimental treatment, and filled three vials per replicate for ^{14}C analysis. The vials from five of the six replicates were incubated for 1 h at three different light levels: darkness, 50, and 100 µmol photons m^{-2} s^{-1}. The three vials of the remaining replicate were used for a 1 h incubation in darkness, a 1 h incubation at 100 µmol photons m^{-2} s^{-1}, and one T_0 measurement with sampling immediately upon addition of the ^{14}C – bicarbonate. This T_0 measurement was

used as a quality control of our ^{14}C analysis, to verify that the measured ^{14}C incorporation was zero at T_0.

For the ^{14}C analysis, a 100 µl sample was transferred to 4 ml Ultima Gold scintillation cocktail (Revvity, Groningen, Netherlands) and total activity was immediately counted in a scintillation counter (‘*DPM total*’ sample). Subsequently, 2 ml of 10% glacial acetic acid in 70% methanol were added to the remaining culture to remove inorganic carbon. Vials were then dried overnight at 60°C until all liquid had evaporated. Residues were re-dissolved in 2 ml of MQ and 10 ml of scintillation cocktail was added for counting in the scintillation counter (‘*DPM fixed’* and *‘DPM fixed dark’*). DPM values obtained were converted into carbon fixation rates (C_{FIX}, in pg C cell^{-1} h^{-1}) as follows:

$$C_{FIX} = \frac{\left(\frac{DPM_{fixed} - DPM_{fixed\ dark}}{DPM_{total}}\right) \times \left(\frac{Total\ DIC}{\Delta t}\right) \times 1.05}{Ochromonas\ abundance} \qquad \text{(Eq. 4)}$$

where 1.05 is the radioisotope discrimination factor, and Total DIC was obtained from the DIC samples taken from each replicate at the day of the physiological experiments. ‘*DPM fixed*’ at T_0 was compared to ‘*DPM fixed dark’* to correct for scintillation activity in the dark incubation samples.

Analysis of cell clumping

Estimation of the extent of cell clumping was done through analysis of the cytograms of each replicate, over the last three transfers. Each cytogram of red fluorescence vs forward scatter was gated twice. One gate (‘singlets’) included only the main cluster of cells which corresponds with single *Ochromonas* cells and contained the majority of events. This gate was used to calculate the mean red fluorescence of that *Ochromonas* sample. The second gate was less stringent and included also events on the diagonal, with higher red fluorescence and forward scatter intensity (‘all *Ochromonas*’). Using a python package, FlowCal[2], the red

fluorescence of each event in the 'all *Ochromonas*' gate was extracted, and divided by the mean 'singlets' red fluorescence value in order to estimate the number of cells in each event. The mean number of cells per event was then calculated by summing all the estimated cell numbers per event across all events in a sample followed by division by the total number of events in the sample. For each replicate, this value was averaged over the last three transfers. While this procedure gives a quantitative estimate of cell clumping, we note that it might not reflect accurate quantification of cells per event as these might not scale linearly with the fluorescence signal detected by flow cytometry.

References:

1. Marie D, Rigaut-Jalabert F, Vaulot D. An improved protocol for flow cytometry analysis of phytoplankton cultures and natural samples. *Cytometry A* 2014;**85**:962–8. https://doi.org/10.1002/cyto.a.22517

2. Castillo-Hair SM, Sexton JT, Landry BP *et al.* FlowCal: A user-friendly, open source software tool for automatically converting flow cytometry data from arbitrary to calibrated units. *ACS Synth Biol* 2016;**5**:774–80. https://doi.org/10.1021/acssynbio.5b00284

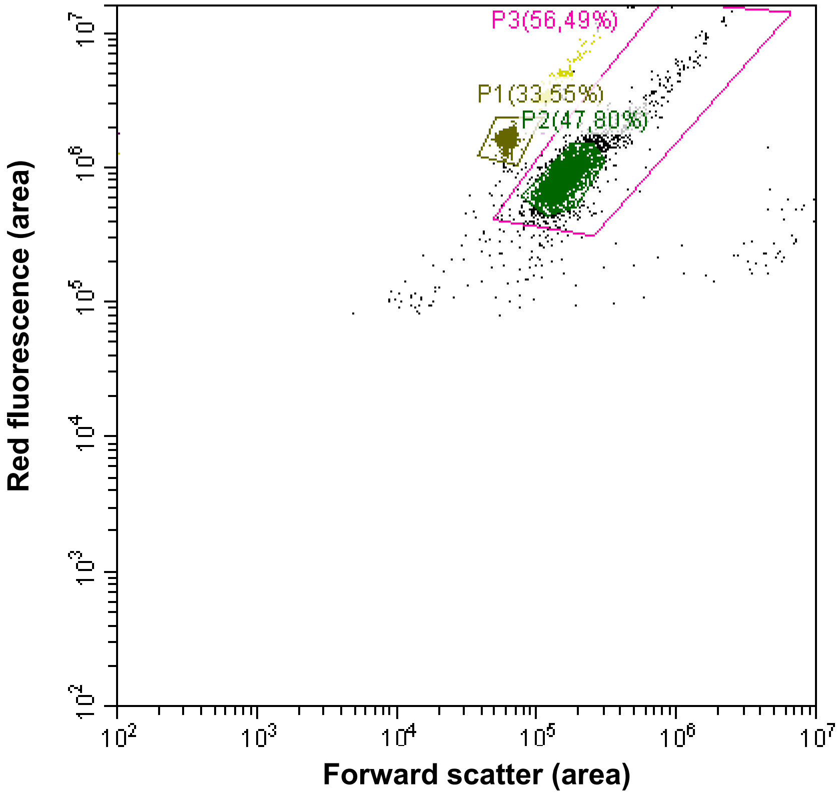

Supplementary figure S1. An example of a raw cytogram from *Ochromonas* counts on the flow cytometer. The sample, of CCMP1393 replicate from the last day of the experiment, was triggered on red fluorescence (690/50) and the following populations were gated based on their red fluorescence (y axis) and their forward scatter (x axis): gate P1 – singlets of red fluorescent beads added to the sample prior to running, gate P2 – singlets of the *Ochromonas* population, gate P3 – all *Ochromonas* cells, including events containing more than one cell.

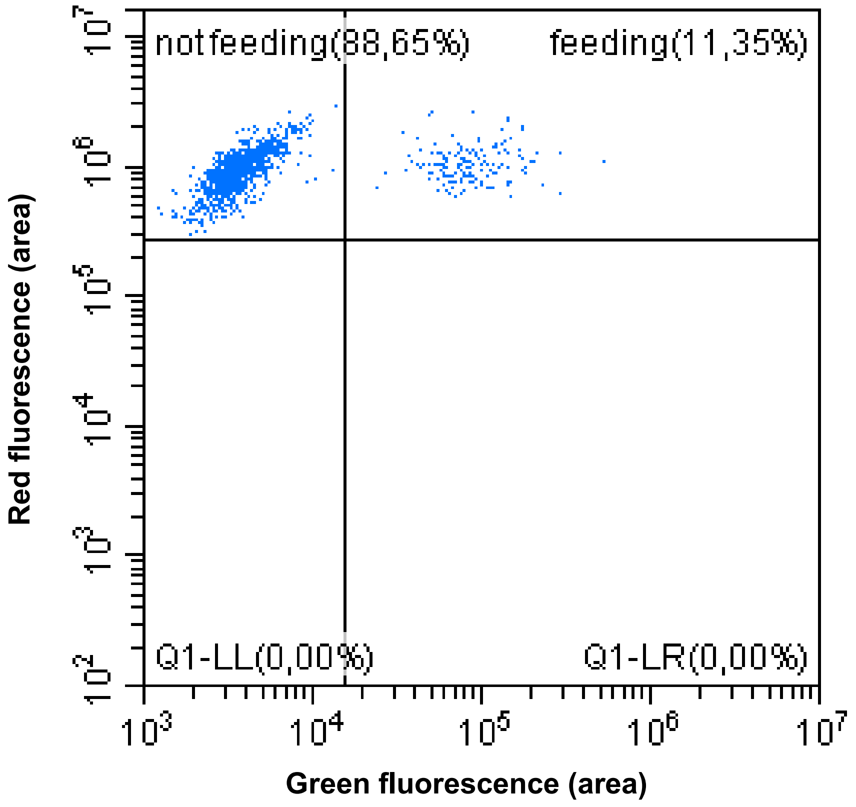

Supplementary figure S2: An example of a cytogram from the grazing assay. *Ochromonas* population (gated based on Fig S1, P2 gate) is plotted based on the red and green fluorescence properties. The population is then separated based on the green fluoresence properties: The population with the low green fluorescence is termed "not feeding" while the population with higher green fluorescence is termed "feeding"

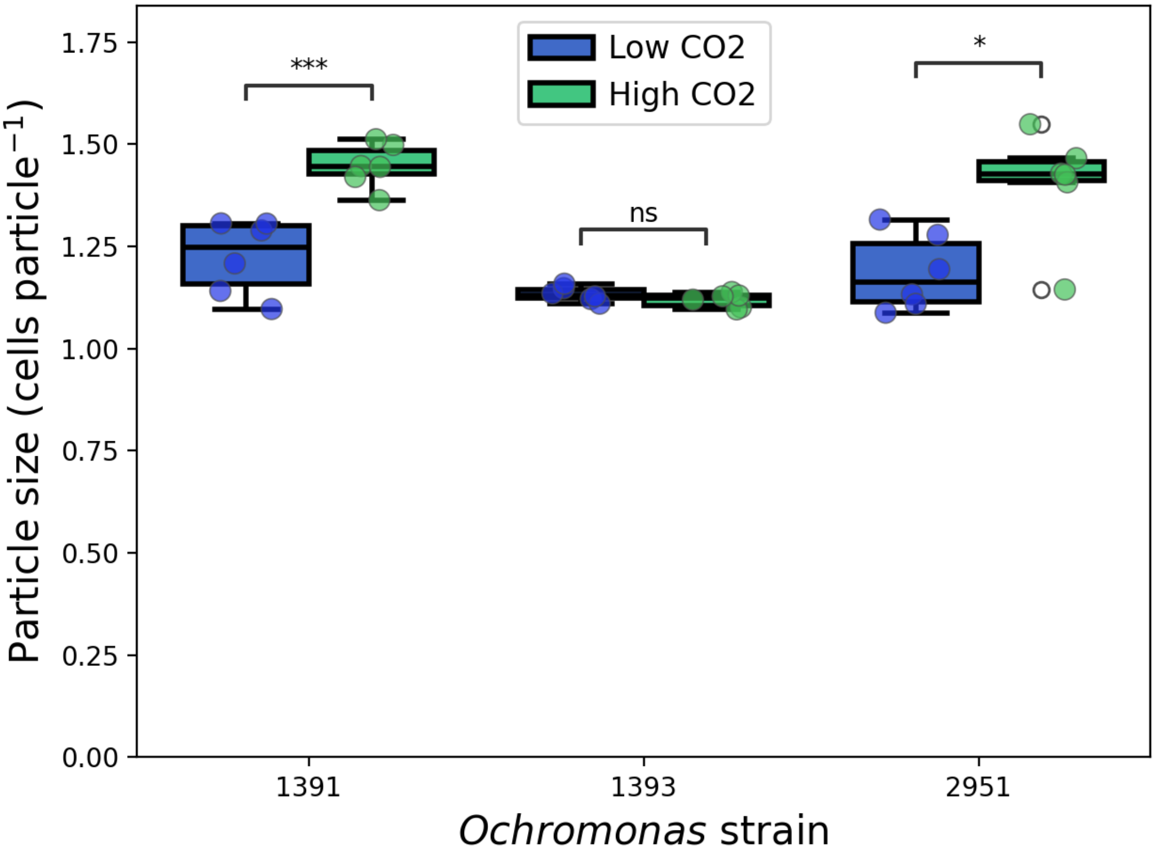

Supplementary figure S3: Estimated number of cells per flow cytometric event (particle) in samples of the three *Ochromonas* strains under Low and High CO_2 treatments. Asterisks indicate siginficant differences between treatments (Welch's *t*-test: $^*p < 0.05$, $^{**}p < 0.01$, $^{***}p < 0.001$).

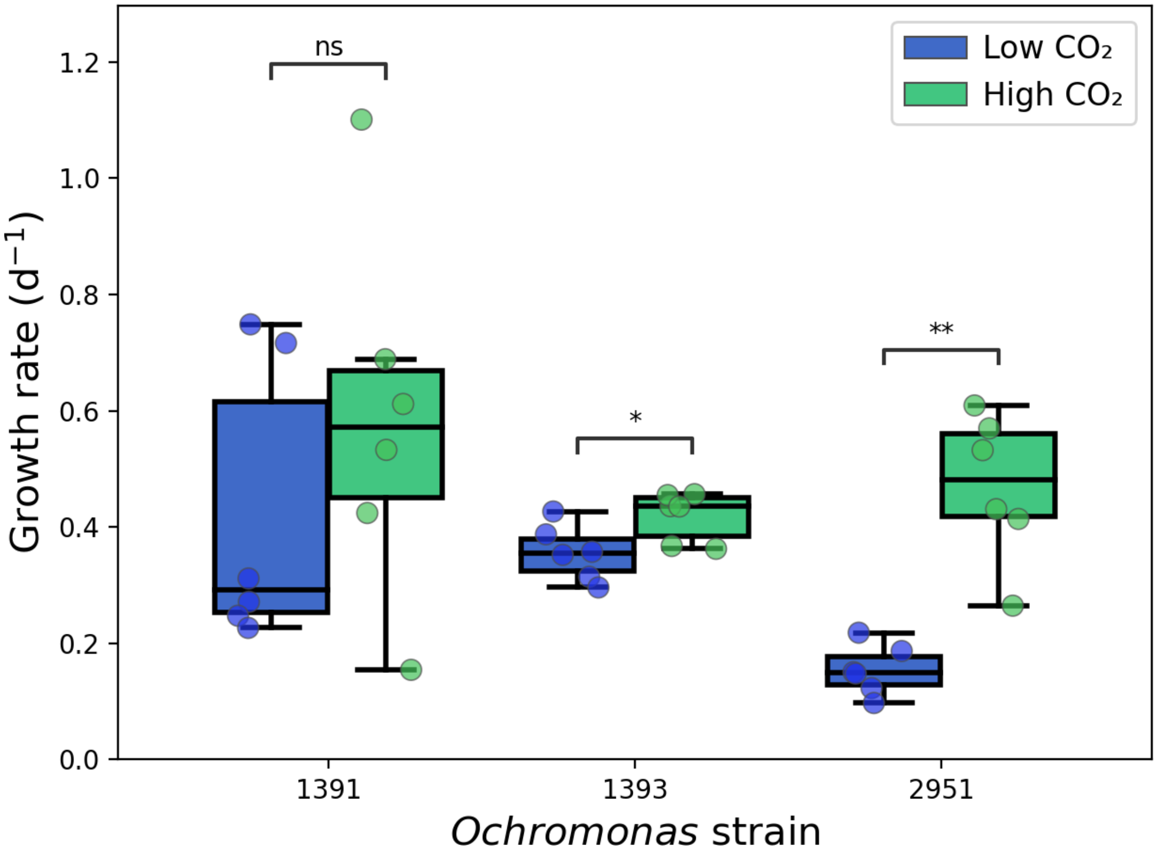

Supplementary figure S4: Specific growth rate of the three *Ochromonas* strains in the Low CO_2 and High CO_2 treatments, based on adjusted flowcytometric cell counts, to account for potential clumping. Asterisks indicate significant differences between treatments (Welch's *t*-test: *$p < 0.05$, **$p < 0.01$, ***$p < 0.001$).

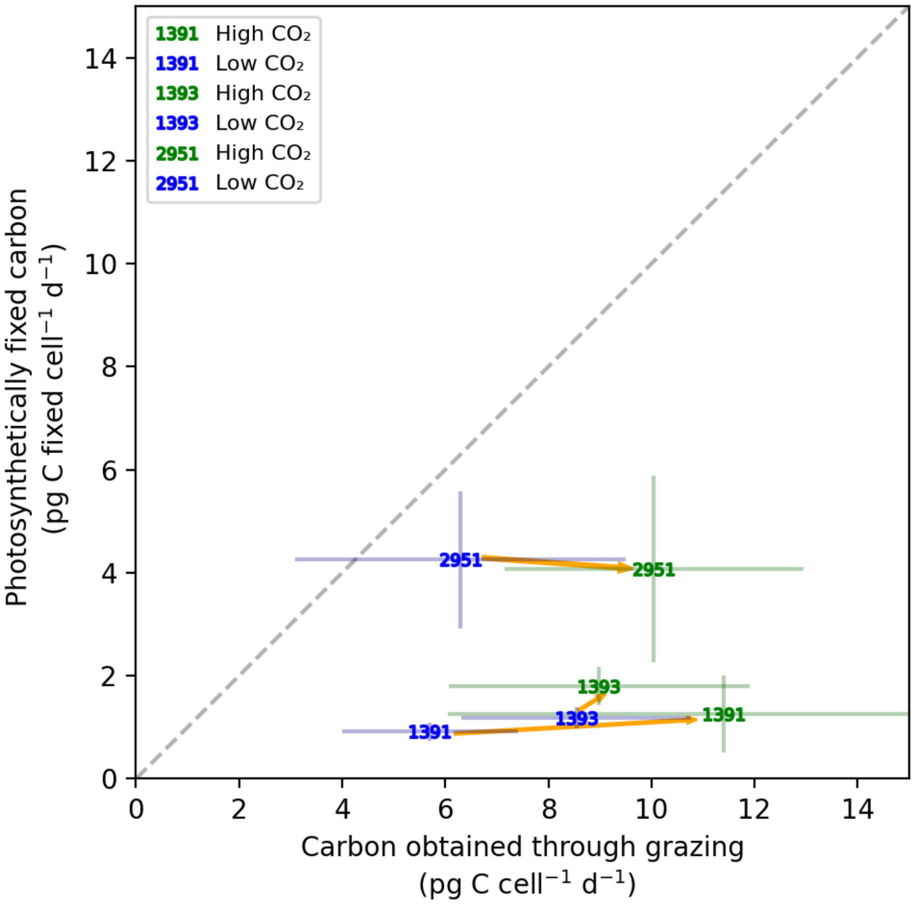

Supplementary figure S5: The balance between phototrophic and heterotrophic carbon acquisition for the three *Ochromonas* strains in the low CO_2 and high CO_2 treatments. The amounts of carbon either obtained from grazing (x-axis) or from photosynthetic carbon fixation (y -axis) per day are extrapolated from measured hourly rates (Figure 3). Error bars represent standard error of the mean. The dashed grey line is the y=x line, at which phototrophic and heterotrophic carbon acquisition are equal. Orange arrows are drawn from the low to the high CO_2 treatment of the same strain.

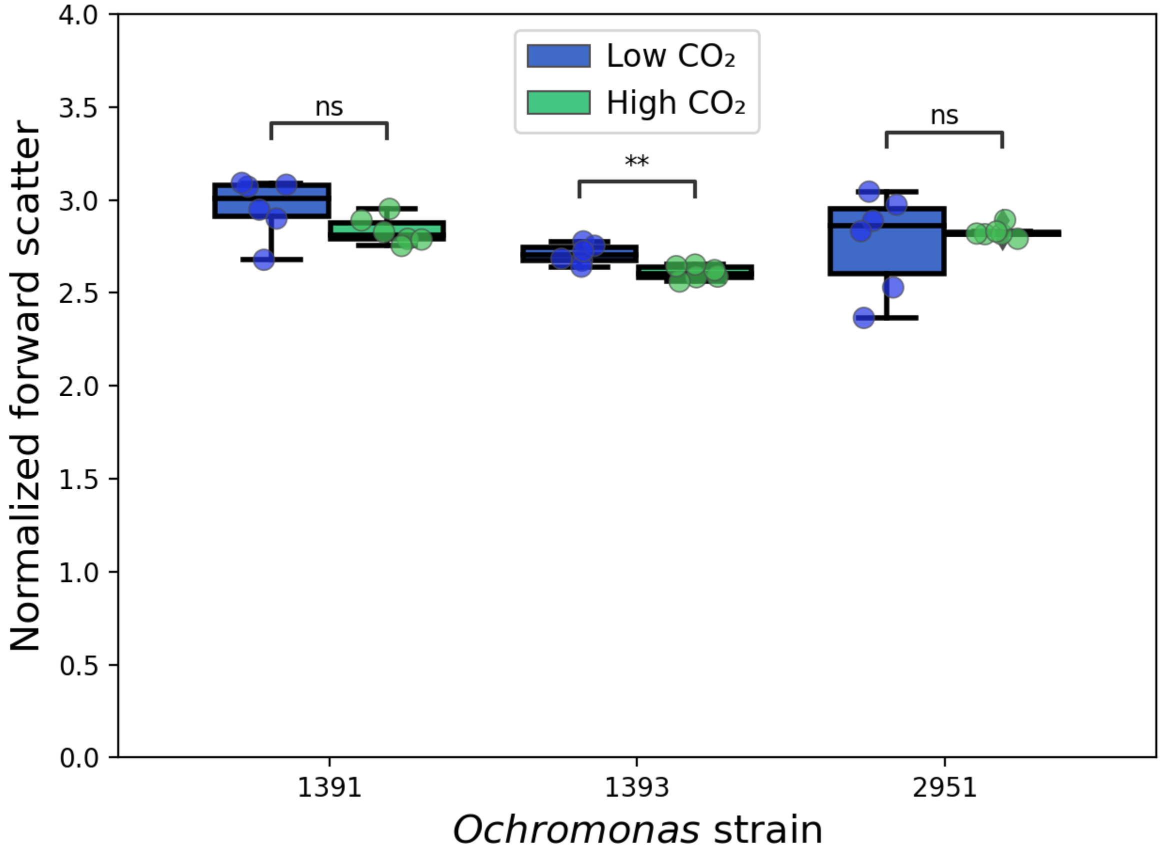

Supplementary figure S6: Relative forward scatter as a proxy for cell size of the three *Ochromonas* strains in the Low CO_2 and High CO_2 treatments, measured by flow cytometry. Asterisks indicate significant differences between treatments (Welch's *t*-test: *$p < 0.05$, **$p < 0.01$, ***$p < 0.001$).

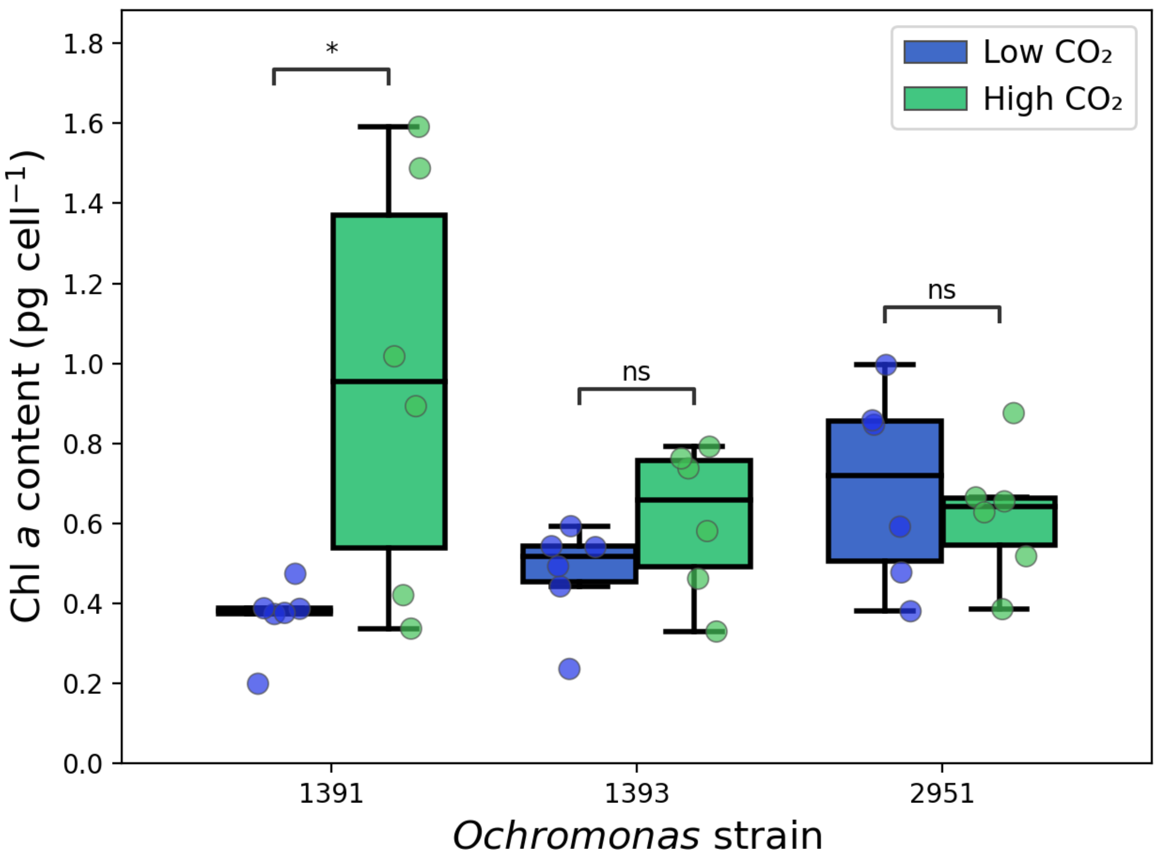

Supplementary figure S7: Chlorophyll *a* content of the three Ochromonas strains under Low and High CO_2 treatments. Asterisks indicate significant differences between treatments (Welch's *t*-test: *$p < 0.05$, **$p < 0.01$, ***$p < 0.001$).

Table S1: Carbonate chemistry of the *Ochromonas* experiments and their controls. Data show mean ± SD (n=6), where each replicate was averaged over the last four transfers. The data are obtained from two consecutive experiments: the first experiment (e1) included *Ochromonas* CCMP2951 and CCMP1391, and the second experiment (e2) included *Ochromonas* CCMP 1393; both experiments included controls without *Ochromonas*. Asterisks in parentheses indicate significant differences of the indicated carbonate chemistry parameter between the low and high CO_2 treatments (Welch's *t*-test: *$p < 0.05$, **$p < 0.01$, ***$p < 0.001$).

**Strain**	**Treatment**	**dissolved CO_2 (ppm)**	**pH**	**DIC (μmol L^{-1})**	**Alkalinity (μEq L^{-1})**
**CCMP 1391**	Low CO_2 (e1)	323 ± 34(***)	8.31 ± 0.01(***)	2266 ± 8(*)	2653 ± 42(***)
**CCMP 2951**	Low CO_2 (e1)	342 ± 74(***)	8.30 ± 0.07(***)	2247 ± 32(**)	2632 ± 36(**)
**CCMP 1393**	Low CO_2 (e2)	362 ± 6(***)	8.32 ± 0.01(***)	2585 ± 12(**)	3013 ± 14(***)
**Control**	Low CO_2 (e1)	335 ± 7(**)	8.30 ± 0.01(**)	2285 ± 72	2663 ± 83(*)
**Control**	Low CO_2 (e2)	402 ± 12(***)	8.28 ± 0.01(***)	2585 ± 71	2976 ± 82
**CCMP 1391**	High CO_2 (e1)	867 ± 46(***)	7.92 ± 0.02(***)	2334 ± 43(*)	2505 ± 40(***)
**CCMP 2951**	High CO_2 (e1)	890 ± 53(***)	7.91 ± 0.02(***)	2370 ± 8(**)	2540 ± 8(**)
**CCMP 1393**	High CO_2 (e2)	783 ± 34(***)	8.02 ± 0.01(***)	2653 ± 39(**)	2890 ± 42(***)
**control**	High CO_2 (e1)	983 ± 135(**)	7.87 ± 0.06(**)	2342 ± 47	2494 ± 63(*)
**control**	High CO_2 (e2)	991 ± 10(***)	7.92 ± 0.01(***)	2696 ± 67	2881 ± 75

Table S2: Statistical comparison of the low CO_2 treatment versus high CO_2 treatment for each of the measured experimental and physiological parameters using the Welch's *t*-test. Degrees of freedom are mentioned once per parameter as they were the same for all strains. Significant differences ($p<0.05$) are indicated in bold.

**Strain**	***1391***		***1393***		***2951***		
**Parameter**	t statistic	p	t statistic	p	t statistic	p	df
**pCO_2**	21.26	**1.2×10^{-9}**	30.03	**3.9×10^{-7}**	14.77	**1.2×10^{-7}**	5
**pH**	-18.54	**1.1×10^{-6}**	-43.80	**7.9×10^{-10}**	-12.19	**1.8×10^{-5}**	5
**DIC**	3.79	**0.0002**	4.13	**0.0063**	8.87	**0.0002**	5
**alkalinity**	-7.04	**4.8×10^{-5}**	-6.84	**0.0004**	-6.17	**0.0011**	5
**Growth Rate**	0.76	0.4645	2.40	**0.0375**	6.26	**0.0006**	**5**
**Photosynthetic C fixation**	1.10	0.3175	3.46	**0.0091**	0.20	0.8434	5
**Slope of C fixation**	0.19	0.8608	2.74	**0.0283**	1.46	0.1938	4
**Grazing rate**	2.48	**0.0476**	0.29	0.7747	2.12	0.0598	5
**Relative red fluorescence**	6.62	**0.0004**	3.11	**0.0112**	2.89	**0.0163**	5
**Fucoxanthin/Chla**	1.62	0.1433	1.14	0.2842	4.01	**0.0095**	5
**b-carotene/chla**	0.18	0.8598	0.60	0.5613	4.20	**0.0050**	5
**VAZ/chla**	2.69	**0.0350**	0.07	0.9439	4.50	**0.0046**	5
**Particle size (cells $particle^{-1}$)**	5.17	**0.0007**	1.46	0.1741	3.21	**0.0109**	5
**Growth rate (counts adjusted to particle size)**	1.02	0.334	2.42	**0.0365**	5.79	**0.0011**	5
**Forward scatter**	1.78	0.1172	3.90	**0.0036**	0.50	0.630	5
**Cellular Chlorophyll a content**	2.74	**0.0386**	1.47	0.1752	0.59	0.5716	5
